# Changes in Physical Fitness and Program Utilization During Professional Military Education

**DOI:** 10.1093/milmed/usag155

**Published:** 2026-04-08

**Authors:** Russell K Lowell, Daniel J Lawson, Katherine A Frick, Nicholas C Bordonie, Katie G Clouse, Cameron D Ott, Harrison C Dimick, Nick R Mauldin, Christopher B Mobley, JoEllen M Sefton

**Affiliations:** Warrior Research Center, School of Kinesiology, Auburn University, Auburn, AL 36801, United States; Department of Kinesiology, University of Alabama, Tuscaloosa, AL 35401, United States; Warrior Research Center, School of Kinesiology, Auburn University, Auburn, AL 36801, United States; Fitomics, Homewood, AL 35209, United States; Warrior Research Center, School of Kinesiology, Auburn University, Auburn, AL 36801, United States; Department of Kinesiology, Mississippi State University, Mississippi State, MS 39762, United States; Warrior Research Center, School of Kinesiology, Auburn University, Auburn, AL 36801, United States; Warrior Research Center, School of Kinesiology, Auburn University, Auburn, AL 36801, United States; Warrior Research Center, School of Kinesiology, Auburn University, Auburn, AL 36801, United States; Warrior Research Center, School of Kinesiology, Auburn University, Auburn, AL 36801, United States; Warrior Research Center, School of Kinesiology, Auburn University, Auburn, AL 36801, United States; Warrior Research Center, School of Kinesiology, Auburn University, Auburn, AL 36801, United States; Warrior Research Center, School of Kinesiology, Auburn University, Auburn, AL 36801, United States

## Abstract

**Introduction:**

Professional Military Education (PME) programs place significant time demands on service members, potentially limiting their ability to maintain physical readiness. This study examined changes in fitness across the PME academic cycle, adherence to Tailored Performance Plans (TPP), and the influence of behavioral and environmental factors on physical fitness.

**Materials and Methods:**

Participants (*n* = 238) completed comprehensive fitness assessments at the beginning and end of a 10-month PME program. In between, they were offered optional TPPs involving 1-on-1 consultations and fitness support. Survey data were collected through a self-report demographic questionnaire.

**Results:**

Body fat percentage (β  =  2.38 ± 0.21%, *P *< .001), fat mass index (β  =  0.65 ± 0.09 kg m^−2^, *P *< .001), flexibility (β  =  1.25 ± 0.25 cm, *P *< .001), and plank (β  =  7.99 ± 2.83 s, *P *= .005) increased following the program, whereas fat-free mass index (β = −0.39 ± 0.08 kg m^−2^, *P *< .001), vertical jump height (β = −0.79 ± 0.29 cm, *P *= .007), and estimated VO_2max_ (β = −0.88 ± 0.22 ml kg^−1^ min^−1^, *P *< .001) declined. TPP participation did not significantly alter these changes. However, a time × TPP interaction emerged for normalized grip strength (β  =  0.74 ± 0.31 kg/m^2^, *P *= .019), indicating maintenance of strength among TPP users although non-users declined. No dose-response relationships were detected between TPP session count and fitness changes.

**Conclusion:**

BOLT participants demonstrated improvements in flexibility and core endurance but reductions in muscle mass, aerobic fitness, and explosive power. TPP engagement did not meaningfully modify these trends. Findings suggest supplemental TPP participation maintained certain neuromuscular outcomes but did not produce broad performance advantages across domains.

## INTRODUCTION

Military service requires personnel to maintain high levels of physical fitness across multiple domains;^1^ including aerobic capacity, body composition, flexibility, muscular strength, and endurance. These demands underscore the need for comprehensive strategies that integrate health, resilience, and performance assessment across overlapping domains.^2^ All branches of service in the United States have a program designed to improve fitness and wellness, for example the Army’s Holistic Health and Fitness (H2F) and Total Force Fitness (TFF) programs, the Air Force’s Comprehensive Airman Fitness Program (CAF), the Navy’s Navy Operational Fitness and Fueling (NOFFS) and Physical Readiness Program (PFA) and the Marine Corps Total Fitness (MCTF). These programs integrate multiple domains—physical, psychological, behavioral, medical, nutritional, spiritual, and social health^2^—critical for optimizing readiness and sustaining long-term performance. They were originally developed for warfighters and within tactical training and deployment contexts.

Service members engage in professional military education (PME) programs, such as the Army War College and Air Command and Staff College, to prepare for increasingly complex leadership roles. These programs require maintaining physical fitness although managing significant cognitive and psychological demands. PME emphasizes critical thinking, strategic awareness, and adaptability through a rigorous curriculum focused on force development, posture, employment, and multinational interoperability.^3-5^ Program length varies by rank and format, ranging from weeks to years, with intellectual growth prioritized over personal pursuits.^6^ Consequently, limited time for physical training and family activities may lead to declines in health and readiness if not addressed.

Previous research on physical fitness during PME has produced mixed findings. A study at the Norwegian Military Academy reported only a slight increase in BMI (0.4 kg/m^2^) and stable aerobic capacity over three years, suggesting that structured physical training can help preserve fitness.^7^ In contrast, U.S. military medical students experienced a 13% decline in push-up performance and a 9% decrease in 1.5-mile run times, with other studies noting similar reductions in VO_2_max and increases in body fat.^8^^,^^9^ Interventions such as circuit-based training^10^ and health education programs^11^ have demonstrated positive effects on strength, endurance, physical activity, and lifestyle habits. Additionally, higher aerobic fitness has been linked to better academic performance.^9^ Collectively, these findings underscore the importance of integrating structured fitness opportunities into PME environments, where demanding cognitive and academic requirements often limit time for physical activity. Providing such support may help counter negative fitness trends and sustain readiness among service members—the military’s most valuable asset.^12^

The United States Air Force’s (USAF) BOLT Program is a comprehensive strategy for Airman Fitness, encompassing mental, spiritual, physical, and social aspects tailored for Air University on Maxwell Air Force Base. The initiative aimed to enhance the well-being and performance of Air University students and faculty by utilizing structured assessments and personalized Tailored Performance Plans (TPPs). This study evaluated pre-post BOLT health, fitness and cognition changes across the first year in a broad Air Force cohort. Additionally, this study seeks to understand how the implementation of TPPs were utilized during the duration of PME. Lastly, self-reported behavioral and environmental factors were evaluated to assess how TPPs influenced fitness outcomes. This work will support and grow the existing body of literature seeking to understand how fitness initiatives, availability to health and fitness resources, and how behavioral and environmental factors may affect fitness and wellness during PME.

## METHODS

### Participants

Air Command and Staff College at Air University, Maxwell Air Force Base students and faculty completed screenings, with 314 individuals providing research consent and completing baseline testing. Of these, 241 participants attended both pre- and post-assessments (attrition rate = 23.3%). Three individuals attended both sessions but did not complete paired physical performance assessments (pre and post) because of medical profile or injury restrictions and were therefore excluded from longitudinal physical analyses. The final sample included 238 consenting participants eligible for paired physical analyses (females = 69, males = 169; age = 36.91 ± 4.54 years; height = 173.97 ± 9.67 cm; body mass = 82.92 ± 14.55 kg). The youngest (24 years) and oldest (64 years) participants were both part of this civilian group. Including non-military civilian participants helped capture the full range of age and experience within this shared work environment. The sample size was determined by the number of available students and faculty at ACSC consenting to participate. Post hoc simulation-based sensitivity analyses were conducted to evaluate the adequacy of the analytical sample size for the primary mixed-effects and multivariate models (detailed in the Statistical Analysis section). Post-program screening occurred in month 10 of the 10-month program. The screenings completed are previously described^13^ and summarized below. Participants read and signed the informed consent approved by the university (IRB# 24-877) and Air Force Institutional Review Boards (IRB# 24-877 EP 240).

### Screenings

Height was measured using a Seca stadiometer (SECA, Hamburg, Germany). Hydration levels were evaluated (V-Resourcing, Hunan, China), dehydrated participants (Urine Specific Gravity ≥ 1.025) were provided water and retested. Weight and body composition were assessed on hydrated participants using bioelectrical impedance analysis via TANITA 568 Segmental Body Composition Analyzer (Tanita Cooperation, Tokyo, Japan). Body fat percentage (%) was used to derive fat mass (kg) and fat-free mass (kg) from total body weight as previously described.^13^ Previous research has confirmed strong correlations between BIA-derived estimates and laboratory criterion methods (*r* = 0.81-0.98), supporting acceptable criterion-referenced agreement at the group level.^14^ However, individual-level error has been reported to range from approximately 1.8%-18.3%, highlighting the importance of standardized testing procedures.^15^^,^^16^ To minimize measurement variability, participants were tested under consistent conditions including (hydration guidelines, device calibration, and trained technicians).

The sit-and-reach test (cm) was used to assess lower back and hamstring flexibility.^17^ Participants sat on the floor, extended their legs and their feet against the SRT box, and reached forward, pushing a bracket as far as possible. The length of the bracket moved was recorded after three testing trials. The sit-and-reach test has demonstrated strong test–retest reliability across diverse age groups (*r* typically > 0.70), with values up to 0.98 reported in large samples and acceptable stability in adult populations.^18^^,^^19^

The Fit Light Trainer (FITLIGHT Sports Corp., Aurora, ON, Canada) was used to assess cognitive response time. Participants deactivated the lights as quickly as possible by tapping the sensor as they illuminated. A familiarization trial (10 hits) followed by three test trials (30 hits each) calculated the average response time (seconds) across the three trials. Previous research has demonstrated good test–retest reliability for choice reaction time, a function of overall response time, when assessed using the FitLight Trainer (intraclass correlation coefficient, ICC = 0.80).^20^

Lower body power was assessed using a countermovement jump on a Leonardo Mechanography force platform (Leonardo Mechanograph, Novotec Medical GmbH, Pforzheim, Germany). The platform sampled both feet at 1,000 Hz. The countermovement jump protocol included two practice jumps and two maximum jumps. Peak countermovement jump height was recorded (cm). Force plate-derived countermovement jump height is considered the gold standard for assessing lower-body demonstrates excellent within-session reliability, with ICC = 0.95-0.96 and coefficients of variation (CV = 3.6%).^21^

Grip strength was measured using an electronic hand dynamometer (Baseline 12-0286 Digital Smedley Dynamometer, Fabrication Enterprises Inc., Elmsford, NY, United States). Participants performed a maximal five-second isometric contraction although maintaining a 90° elbow angle, alternating hands in sequential order with the highest value used for analysis. The maximal value was normalized by height squared (kg/m^2^).^22^ Handgrip strength testing has demonstrated excellent reliability (ICC ≥ 0.90) and high inter-rater objectivity (*r* = 0.97) under standardized procedures.^23-25^

Overall force-producing capabilities were evaluated using the Isometric Midthigh Pull with a portable isometric and a wooden platform with rubber grips and a straight bar attachment (Peak Force Systems, Mesquite, TX, United States). Participants performed three submaximal pulls at 50%, 65%-75%, and 80%-90% of their perceived maximal effort. Two maximum effort pulls followed, with 30 seconds between trials. Peak force was recorded in kilograms (kg) for all maximal trials. Studies have shown the isometric mid-thigh pull is time-efficient, induces minimal fatigue, and may present a safer alternative to dynamic 1RM testing. Additionally, isometric midthigh pull peak force is strongly correlated with 1RM back squat performance (*r* ≥ 0.96).^26^^,^^27^

A maximum forearm plank was used to assess muscular endurance. Results were compared to normative benchmarks from a general population chart. The prone plank test has demonstrated excellent test–retest reliability over repeated testing sessions (ICC = 0.915) and evidence of construct validity across age and activity levels.^28^

Participants completed a 6-minute walk/run test to estimate maximal oxygen consumption.^29^ They walked, ran, or combined as needed to cover the maximal distance possible. Instructors monitored their time during each 196.9-m lap and calculated the total distance covered. Timed field-based run/walk tests have demonstrated high reliability and validity for estimating aerobic capacity. For example, the Cooper 12-minute run test has shown strong test–retest reliability (*r *≈ 0.90) and strong associations with laboratory-measured VO_2max_. The 6-minute walk/run protocol used in this study represents a similar practical field-based assessment administered under standardized conditions.^30^

Participants completed a demographic and wellness survey developed for the BOLT Program at Air University. Participants ranked the top three factors most influencing their physical activity and indicated whether each factor had a positive, neutral, or negative impact. Positive was defined as good, useful, supportive, or favorable. Negative was defined as bad, lacking, unsure, doubtful. The listed factors included time, health, facilities, current fitness level, command climate, family demands, work demands, and peer pressure.

### Tailored Performance Plans

TPPs were offered to all ACSC cohort students and staff as voluntary, individualized consultations focused on health, wellness, and fitness in person or virtually. Unlimited scheduled or walk-in appointments were available covering a wide a range of topics, including screening review, exercise technique, stress reduction and fitness planning. The number of sessions, location, and context of each session was recorded.

### Statistical Analysis

Descriptive statistics were computed for all pre- and post-program variables. Data are reported as mean ± standard deviation (SD). Normality of the change scores was evaluated using the Shapiro–Wilk and Anderson–Darling tests. Several variables deviated from normality, however, the primary analytical models (linear mixed-effects and multivariate multiple regression) are robust to these violations. Non-parametric Wilcoxon signed-rank tests were also conducted as sensitivity analyses, and results were largely consistent with parametric tests.

Two sets of linear mixed-effects models were conducted to evaluate changes across time and the influence of the TPP program. The first model included timepoint (pre vs. post) as a fixed effect, with a binary indicator for TPP participation (“yes” or “no”) and its interaction with timepoint to test whether individuals who participated in TPP experienced different outcomes than non-participants. The second model included a continuous measure of TPP session count (dose) and its interaction with timepoint to examine potential dose-response effects. All models controlled for sex and age, with participant ID modeled as a random intercept. Fixed effects, standard errors, degrees of freedom, t-statistics, and p-values were extracted, and model fit was compared using Akaike Information Criterion (AIC).

Survey data were analyzed to identify perceived facilitators and barriers to fitness progress. Participants selected up to three personal or contextual factors and indicated whether each had a positive or negative influence. Frequencies and percentages were calculated for each response. Survey responses were recoded as ordinal dummy variables (–1 = negative, 0 = neutral or not selected, 1 = positive) to enable quantitative modeling and merged with post-BOLT program fitness outcomes.

A multivariate multiple linear regression (MMR) was conducted with a matrix of continuous fitness outcomes (BMI, FMI, FFMI, Sit-and-Reach, Vertical Jump, Normalized Grip Strength, relative IMTP, Plank, and estimated VO_2max_) regressed simultaneously on the set of survey predictors. This approach evaluated the unique contribution of each perceived factor across multiple interrelated outcomes although accounting for shared variance among the dependent variables.^31^ Standardized coefficients (β), standard errors, 95% confidence intervals, and p-values were reported for each predictor–outcome relationship. Predictors and outcomes were standardized (z-scores) to allow direct comparison of effect magnitudes across variables. Survey items were coded as ordinal contrasts, standardized coefficients represent the strength and direction of association between a ± 1 SD change in the predictor score and a ± 1 SD change in the outcome variable. Model diagnostics indicated all key assumptions for MMR were reasonably satisfied. The Variance Inflation Factor (VIF) indicated minimal multicollinearity (VIF = 1.03-1.56), and no evidence of nonlinear relationships was detected (Ramsey RESET *P* > .05). Homoscedasticity was supported by non-significant Breusch–Pagan (*P* > .05) and Non-Constant Variance tests (*P* > .05).^32^^,^^33^ Shapiro–Wilk tests suggested mild deviations from normality for several outcomes, visual inspection of Q–Q plots showed no substantial departures and model robustness was maintained. Cook’s distance and leverage values confirmed the absence of influential outliers.^34^

Simulation-based sensitivity analyses were conducted to evaluate the adequacy of the analytic sample size^35^^,^^36^ in this fixed cohort study. In mixed-effects models, simulations preserved the estimated random-intercept and residual-variance structures. Power to detect small overall pre–post effects was consistently high (≥0.85 across outcomes and frequently >0.95). In contrast, power to detect small interaction effects (time × TPP) ranged from approximately 0.15 to 0.80 depending on outcome, increasing substantially for moderate and large effects and approaching 1.00 in most cases. For MMR, simulations preserved the observed predictor structure and residual covariance matrix, with power estimated for the global Pillai trace test. Power was 0.99 at the observed effect magnitude, remained above 0.80 when effects were reduced to 75% of the observed magnitude, and declined to 0.37 when effects were reduced to 50%, indicating adequate sensitivity to detect moderate-to-large multivariate effects but limited sensitivity for small effects.

All statistical analyses were performed in R (version 4.4.2) and RStudio Pro (2024.12.1 Build 563). Data wrangling and visualization were performed using the tidyverse R package, which includes dplyr, tidyr, ggplot2, purrr, and forcats. Mixed-effects models were estimated using lme4 and lmerTest, with model diagnostics supported by the car and performance packages. Data summaries and regression outputs were organized using broom. Visualization enhancements included ggpubr, ggdist, gghalves, and ggtext packages. Normality checks were carried out using nortest, and traditional statistical procedures were implemented using base stats. Simulation-based sensitivity analyses were implemented for mixed-effects models via custom Monte Carlo procedures in lme4 and lmerTest; the simr package served as a supporting framework for simulation-based power workflows. MMR was conducted using base R modeling functions (stats) and multivariate simulation procedures from MASS (mvrnorm).

## RESULTS

Subject characteristics and fitness descriptives are reported in the Appendix (Table S1). Sample sizes for each outcome variable reflect complete-case paired data (non-missing Pre and Post values). As a result, the analytic sample size varies slightly by outcome, with a maximum of 230 depending on variable-specific missingness. Fitness change scores are visualized in Figure 1.

**Figure 1.**
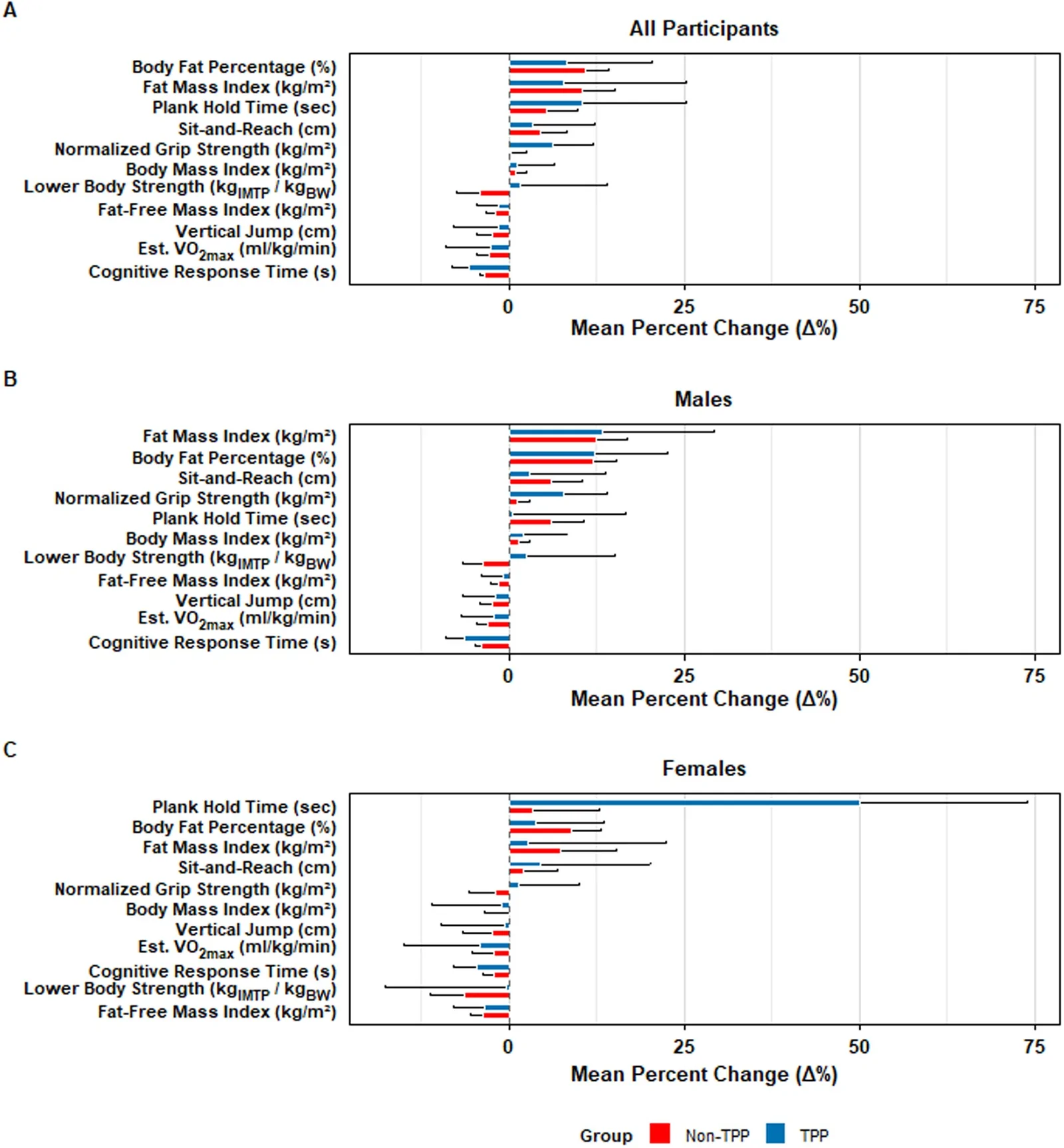
Mean percent change (Δ%) in fitness outcomes from pre- to post-program for tailored performance plan (TPP) and non-TPP participants. Panels A, B, and C display results for all participants, males, and females, respectively. Fitness outcomes are ordered by mean change, and error bars represent standard error (S.E.) for each group. TPP and Non-TPP participants are compared within each outcome to highlight potential differences in response. Relative lower body strength is represented as the ratio of IMTP force (kg_IMTP_) to body weight (kg_BW_). Improvements are indicated by rightward bars, and a vertical dashed line denotes zero change.

### Tailored Performance Plan Utilization

A total of 104 TPP sessions were completed between October 2024 and April 2025. Utilization was highest in October 2024, with 51 sessions conducted among 29 unique participants, including 22 individuals who returned for multiple sessions. TPP engagement declined in subsequent months despite positive feedback, with session counts ranging from 1 to 16 per month (Figure 2). Repeat participation remained evident, particularly in January and March 2025, where the number of repeat participants exceeded the number of unique monthly participants.

**Figure 2.**
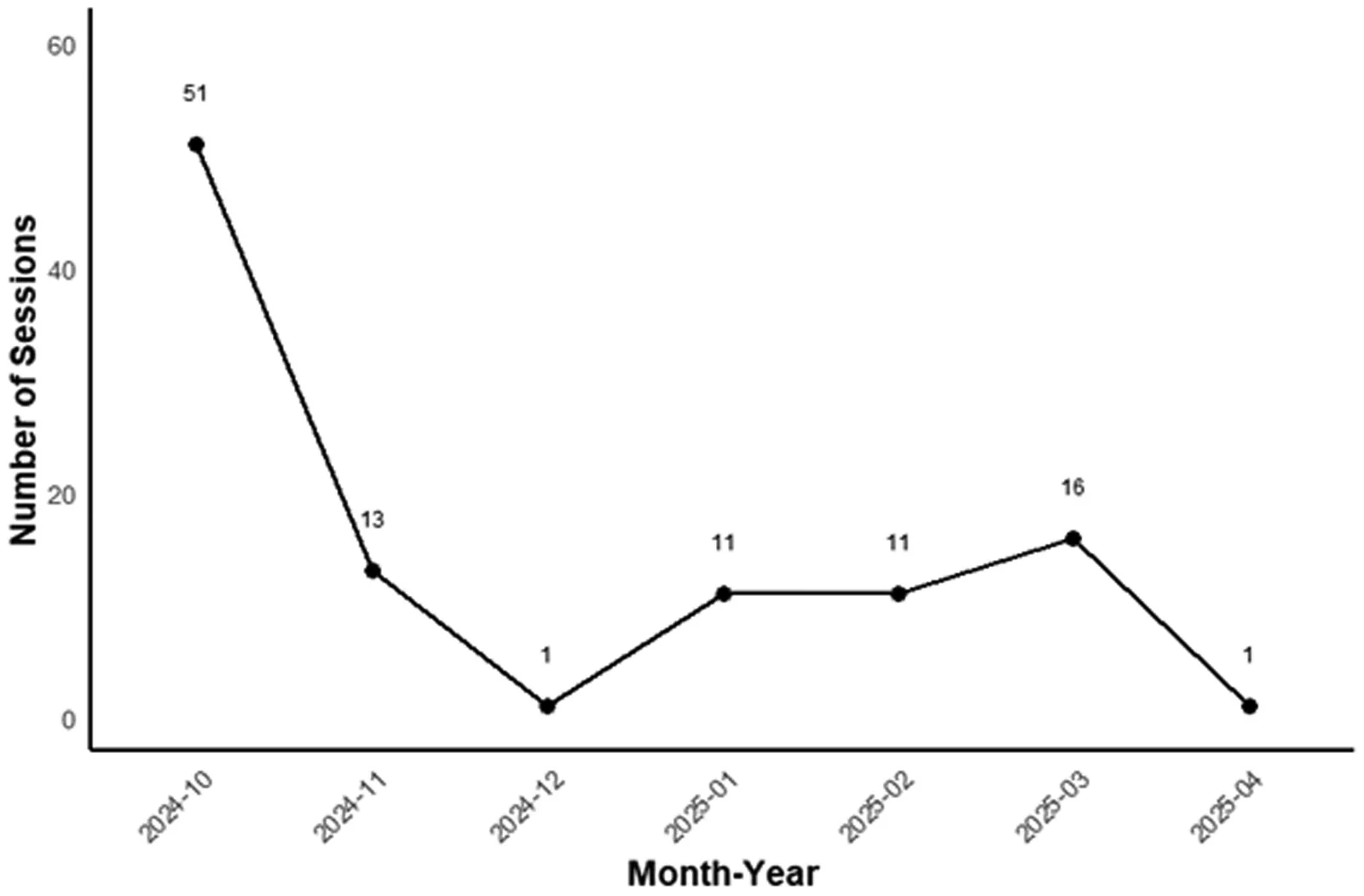
Monthly tailored performance plan (TPP) utilization from October 2024 through April 2025. The line represents the total number of TPP sessions conducted each month, with data labels indicating exact session counts.

### Pre-Post Changes and TPP Usage

Most fitness and performance measures changed significantly from pre- to post-testing across all participants (Table 1). Participant characteristics and descriptive statistics for TPP and Non-TPP use can be found in the Appendix (Tables S2-S5).

**Table 1.** Pre-Post Changes in Fitness Metrics Across All BOLT Participants

Variable	MD	*P*-value
Body mass index (kg m^2^)	0.20	*P *= .180
Body fat percentage (%)	2.41	*P* < .001
Fat mass index (kg m^2^)	0.67	*P* < .001
Fat-free mass index (kg m^2^)	−0.39	*P* < .001
Sit-and-reach test (cm)	1.37	*P* < .001
Cognitive response time (sec)	−1.27	*P* < .001
Vertical jump height (cm)	−0.81	*P* = .008
Grip strength (kg/m²)	0.15	*P* = .143
Isometric midthigh pull (kg_IMTP_/kg_WT_)	−0.01	*P* < .001
Estimated VO_2max_ (ml kg^-1^ min^-1^)	−0.85	*P* < .001

All participants, *n* = 238. MD = Mean Difference (post—pre). Positive values indicate increases from pre- to post-assessment, and negative values indicate decreases.

Mixed-effects models were used to examine pre–post changes in fitness outcomes and whether these changes differed between TPP users and non-users (Model 1), as well as to assess dose–response effects of TPP session count (Model 2). Results are reported in the Appendix (Table S6).

In Model 1, a significant time × TPP interaction was observed for grip strength (β  =  0.737 kg/m^2^, *P *= .019), indicating relative grip strength declined among non-TPP participants but had a positive effect on TPP users. A potential moderating effect was also observed for cognitive reaction time, with the interaction approaching significance (β = −0.812 sec, *P *= .083). No other significant interaction effects were observed, indicating TPP participation did not meaningfully alter pre–post changes in other outcomes. Across all participants there were significant main effects of time, reflecting overall pre–post changes in multiple ­variables: body fat percentage (β  =  2.405%, *P *< .001), FMI (β  =  0.646 kg m^−2^, *P *< .001), FFMI (β = −0.398 kg m^−2^, *P *< .001), sit-and-reach test (β  =  1.367 cm, *P *< .001), vertical jump height (β = −0.807 cm, *P *= .008), relative lower body strength (β = −0.135 kg_IMTP_/kg_WT_, *P *= .004), plank (β  =  7.061 sec, *P *= .017), and estimated VO_2max_ (β = −0.851 ml kg^−1^ min^−1^, *P *< .001). These changes occurred independently of TPP participation, suggesting consistent pre–post trends across all participants.

In Model 2, which replaced the binary TPP indicator with TPP session count (dose) and its interaction with timepoint, no significant dose–response interactions were observed for any variable. The timepoint × TPP count term was non-significant for all outcomes, including relative grip strength (β  =  0.087 kg/m^2^, *P *= .239) and estimated VO_2_max (β  =  0.095 ml kg^−1^ min^−1^, *P *= .533). Main effects of timepoint remained significant for the same variables as in Model 1, confirming consistent overall pre–post changes regardless of TPP dose.

Across both models, age and sex were consistent covariates. Older participants exhibited higher adiposity, lower aerobic capacity, slower cognitive reaction times, and reduced muscular strength. Males demonstrated greater absolute strength, power, and aerobic fitness, whereas females showed superior flexibility and relative strength when normalized to body weight. These associations remained stable across TPP and non-TPP groups.

### Survey–Fitness Predictive Analysis Results

Descriptive frequencies of survey responses are presented in Table 2. The most reported positive indicators included health (*n* = 65, 10.1%) and time (*n* = 53, 8.2%). The most frequently reported negative indicators were time (*n* = 128, 19.9%), work demands (*n* = 121, 18.8%), and family demands (*n* = 98, 15.2%).

**Table 2.** Survey Responses

Factor	Negative (*N*=)	Positive (*N*=)	Negative (%)	Positive (%)
Time	128	53	19.90	8.20
Work demands	121	18	18.80	2.80
Family demands	98	23	15.20	3.60
Health	26	65	4.00	10.10
Current fitness	8	44	1.20	6.80
Facilities	6	27	0.90	4.20
Peer pressure	2	12	0.30	1.90
Command climate	8	4	1.20	0.60

Positive was defined as good, useful, supportive, or favorable. Negative was defined as bad, lacking, unsure, doubtful.

Several self-reported barriers showed significant associations with standardized fitness outcomes. Health beliefs predicted multiple outcomes: more positive health perceptions correlated with lower BMI (β = –0.128, *P *= .019) and lower fat mass index (β = –0.139, *P *= .011), as well as greater plank hold time (β  =  0.13, *P *= .016) and higher estimated VO_2max_ (β  =  0.11, *P *= .047). Family demands were negatively associated with fat-free mass index (β = –0.127, *P *= .039), suggesting competing responsibilities may limit maintenance of lean mass. Estimated VO_2max_ improvements were predicted by time (β  =  0.160, *P *= .011) and perceived health (β  =  0.107, *P *= .047).

Other predictors, including perceived facility access, command climate, and peer influence, showed no significant relationships across the fitness measures (*P *> .05). Full standardized regression coefficients and 95% confidence intervals appear in Figure S1.

## DISCUSSION

The purpose of this study was to compare pre- and post-fitness changes following implementation of TPPs among professional military personnel in a PME environment and to examine behavioral and environmental factors that may influence fitness outcomes. The results demonstrated declines in fitness outcomes over the academic cycle, decreasing utilization of TPPs likely limited their effectiveness, and negative survey responses to the behavioral and environmental factors were associated with poorer fitness outcomes. These findings suggest external contextual factors, which may significantly influence fitness trends and fitness/wellness program use during PME.

Collectively, the findings suggest the primary barrier to both TPP utilization and fitness maintenance was insufficient time, governed by the academic rigor and competing demands outside and within the PME. The demographic survey indicated 54% of negative survey responses were related to time limitations, work demands, and competing family obligations. Participants often struggled to prioritize physical training within the constraints of a demanding schedule even when provided with individualized fitness resources. This is consistent with previous work recognizing time scarcity as a critical hurdle to exercise adherence in general.^37^^,^^38^

Tailored fitness programming provides individualized guidance in areas specifically requested by participants. However, its success is dependent on consistent utilization and sufficient training dose. TPPs were heavily utilized initially but quickly decreased over time even though feedback on TPP sessions was strongly positive. Low overall utilization of the TPP program limited the ability to evaluate its true effectiveness. These challenges are consistent with larger trends observed in exercise intervention research.^37^ The authors reported dropout rates in exercise studies commonly range from 25% to 50%. Among participants completing the interventions, adherence averages around 66% of the planned program.^37^ In the current study, TPPs were voluntary, which allowed students to partake in appointments whenever their schedule allowed. The self-directed nature of the TPPs may have placed greater responsibility on the individual to allocate time and prioritize fitness. Our results found those individuals participating in TPPs were largely those who were the most fit (green in a red/yellow/green fitness classification based on pre-program fitness screening).^13^ This suggests these participants already had strong fitness and time management habits leading us to believe those individuals may have been able to sustain fitness more easily those adding new fitness programming into their routine although also participating in a new academic program.

The addition of structured guidance, time, or accountability may improve the effectiveness of programs even for those who regularly engage in some level of physical activity or exercise during the academic cycle. A study comparing supervised versus unsupervised training revealed more positive changes in body composition, aerobic fitness, and strength outcomes when programs are guided by a consistent, structured intervention facilitated by a fitness professional rather than relying on individual initiative.^39^ Specifically, the supervised group experienced improvements in lean body mass (approx. 1.3 kg avg. increase), chest press strength (42% increase), peak leg power (6% increase), and VO_2max_ (7% increase), although the self-directed group saw minimal or no gains in these measures.^39^ These findings suggest students and faculty at ACSC may benefit from regular, supervised structured interventions and scheduled fitness time during their time at Air University. Research has also shown fitness retention rates are increased when individuals are required to be in groups or have accountability.^40^ These group-oriented environments can foster consistency, engagement, and an increased sense of shared purpose. However, requiring participation or demanding daily or weekly fitness requirements (e.g., morning physical training) without autonomy may risk unintended consequences, including negative perceptions of exercise, reduced intrinsic motivation, or feelings of punishment. Although physical readiness is already a core value in the military, strengthening its part as a culturally modeled expectation within the PME environment may encourage more consistent engagement and self-accountability.

Future implementations should consider embedding low-burden opportunities (brief check-ins at key points in the academic cycle as a part of the curriculum, etc.) to better align with participants schedules. The BOLT team was always present at the ACSC site and at monthly wellness lunch talks. However, these talks were voluntary, not a part of the curriculum, and saw decreased attendance even with very positive survey reviews. Incorporating more hands-on opportunities such as nutrition sessions, movement techniques in the fitness center, or quick “walk-and-talk” consultations would offer approachable entry points for those who may not seek out a traditional appointment. These changes would not remove the external challenges inherent to the PME environment, but they may better position TPPs as an actionable, integrated resource rather than an optional extra. Activities viewed as being additional to the already heavy workload were seen as unnecessary and not important to superiors.

Negative survey responses regarding behavioral and environmental influences on physical fitness were associated with poorer fitness outcomes. These findings reflect a larger pattern of cumulative stress and competing priorities known to interfere with regular physical training, especially in high-responsibility military populations.^37^^,^^38^ Time constraints were the most frequently reported barrier to physical activity in survey responses. However, although commonly reported, analysis indicated time constraints alone may not fully explain observed fitness changes. In contrast, internal beliefs and social and environmental context revealed stronger and more consistent associations with physical fitness outcomes. This highlights the need to look beyond perceptions of time scarcity and address the hidden behavioral and environmental factors that may influence physical readiness.

Health-related perceptions appeared as one of the most consistent predictors of favorable fitness outcomes, specifically in relation to body composition measures. Participants who reported more positive views of their overall health exhibited lower body mass index, body fat percentage, and fat mass index during post-testing. These perceptions may suggest more positive engagement with physical activity and health related awareness throughout the academic cycle. Prior research supports this interpretation, demonstrating individuals participating in regular physical activity perceive themselves to be in good health and are more likely to maintain healthier lifestyles over time.^41^^,^^42^ Those who perceive their physical fitness in a more positive way may be more motivated to continue to improve their fitness levels. Research suggests older adults with low self-efficacy (the belief their capacity to succeed in a specific situation or task) tend to reduce both the number of activities they engage in and the effort they expend.^41^ This suggests positive health perceptions may reflect higher self-efficacy, leading individuals to pursue more healthy behaviors and expend greater effort to do so. These findings also resonate with the Department of Defense’s Total Force Fitness framework, which emphasizes the interaction of psychological, behavioral, and social factors in shaping operational readiness.^2^ Physical training may not always receive consistent prioritizing within the PME environment. Behavioral readiness influences such as motivation, self-perception, and social support should be considered as catalysts of physical training to prevent this from becoming habitual. Supporting these psychological and behavioral dimensions may be essential for maintaining fitness and readiness in this high-performing, yet time-constrained population.

## CONCLUSION

This study examined physical and fitness changes during the academic implementation of a personalized fitness initiative during PME, where participants faced significant academic and time demands. Despite the accessibility of TPPs, fitness levels generally declined over the academic cycle. Survey results revealed individuals who reported more positive health perceptions and greater confidence in their fitness were more likely to maintain healthier outcomes, whereas commonly reported barriers like time constraints, though frequently mentioned, were not strongly linked to performance changes. These findings suggest fitness interventions must be more than available, perhaps they may need to be actively supported, integrated into the daily structure, and reinforced by leadership. In high-demand environments like PME, success may depend on combining flexible programs with intentional scheduling, accountability, and a culture prioritizing physical readiness as part of professional development.

## Supplementary Material

usag155_Supplementary_Data

## Data Availability

The data underlying this article will be shared on reasonable request to the corresponding author and Air Force approval.

## INSTITUTIONAL CLEARANCE

Publication of this article was approved by the US Air Force Public Affairs office at Maxwell Air Force Base, Alabama.
