# Supplementary material for: Changes in Physical Fitness and Program Utilization During Professional Military Education
Source: Mil Med. 2026 Apr 8;191(11-12):e2458–66. doi: 10.1093/milmed/usag155 (PMC13730447; doi:10.1093/milmed/usag155)
Supplement: Supplementary file 1 [file usag155_supplementary_data.zip › Supplementary materials v2.docx]

**Supplementary Materials**

| **Supplementary Table 1. Participant Characteristics and Descriptive Statistics** | | | | | |
| --- | --- | --- | --- | --- | --- |
| **Variable** | **Sex** | **n** | **Pre- Mean (SD)** | **Post-Mean (SD)** | **MD** |
| Age (yrs) | Male | 165 | 36.81 (± 4.36) | 37.49 (± 4.44) | 0.68 |
|  | Female | 67 | 37.12 (± 4.99) | 37.69 (± 4.06) | 0.57 |
| Height (cm) | Male | 163 | 177.92 (± 7.26) | 177.87 (± 7.88) | -0.05 |
|  | Female | 65 | 164.07 (± 7.66) | 164.02 (± 7.04) | -0.05 |
| Body Mass (kg) | Male | 161 | 86.69 (± 12.72) | 88.08 (± 12.77) | 1.39 |
|  | Female | 65 | 73.57 (± 14.66) | 73.87 (± 14.30) | 0.29 |
| Body Mass Index (kg·m²) | Male | 161 | 27.43 (± 3.85) | 27.82 (± 3.59) | 0.39 |
|  | Female | 65 | 27.42 (± 5.95) | 27.39 (± 5.71) | -0.02 |
| Body Fat Percentage (%) | Male | 161 | 20.25 (± 6.03) | 22.66 (± 6.20) | 2.41 |
|  | Female | 64 | 32.74 (± 7.72) | 35.38 (± 7.72) | 2.65 |
| Fat Mass Index (kg·m²) | Male | 159 | 5.72 (± 2.29) | 6.43 (± 2.11) | 0.71 |
|  | Female | 64 | 9.34 (± 4.26) | 9.96 (± 4.58) | 0.62 |
| Fat Free Mass Index (kg·m²) | Male | 159 | 21.6 (± 2.04) | 21.32 (± 2.86) | -0.29 |
|  | Female | 64 | 18.06 (± 2.09) | 17.42 (± 2.57) | -0.64 |
| Functional Reach Test (cm) | Male | 160 | 39.35 (± 5.71) | 43.39 (± 5.27) | 4.04 |
|  | Female | 66 | 38.2 (± 5.47) | 40.12 (± 5.77) | 1.93 |
| Sit-and-Reach Test (cm) | Male | 159 | 24.77 (± 9.52) | 26.12 (± 9.17) | 1.35 |
|  | Female | 65 | 31.67 (± 8.41) | 32.42 (± 8.71) | 0.75 |
| Cognitive Response Time (s) | Male | 162 | 32.29 (± 2.13) | 30.95 (± 2.25) | -1.34 |
|  | Female | 65 | 33.35 (± 2.49) | 32.51 (± 2.87) | -0.84 |
| Countermovement Jump (cm) | Male | 152 | 41.64 (± 6.27) | 40.42 (± 6.70) | -1.22 |
|  | Female | 61 | 30.32 (± 6.68) | 29.7 (± 6.59) | -0.62 |
| Grip Strength (kg/m²) | Male | 163 | 16.51 (± 2.70) | 16.79 (± 2.60) | 0.28 |
|  | Female | 63 | 12.46 (± 2.40) | 12.29 (± 2.58) | -0.18 |
| Isometric Midthigh Pull (kg) | Male | 128 | 225.6 (± 55.54) | 218.28 (± 55.02) | -7.32 |
|  | Female | 51 | 138.9 (± 35.89) | 131.61 (± 35.89) | -7.29 |
| Plank (s) | Male | 160 | 154.52 (± 59.58) | 160.88 (± 59.12) | 6.36 |
|  | Female | 61 | 121.93 (± 56.43) | 132.21 (± 56.63) | 10.28 |
| Estimated VO_2max_ (ml·kg^-1^·min^-1^) | Male | 151 | 38.22 (± 4.84) | 37.12 (± 4.90) | -1.10 |
|  | Female | 61 | 30.6 (± 4.95) | 29.84 (± 4.20) | -0.76 |
| ***Note:*** *Values are presented as mean (± SD). MD = Mean Difference (post – pre). Positive values indicate increases from pre- to post-assessment, and negative values indicate decreases.* | | | | | |

| **Supplementary Table 2: Participant Characteristics and Descriptive Statistics for TPP Users** | | | | | |
| --- | --- | --- | --- | --- | --- |
| **Variable** | **Group** |  | **Mean (± SD)** | **Mean (± SD)** | **Mean_Diff** |
| Age | All | 27 | 38.41 (± 6.25) | 38.89 (± 6.23) | 0.48 |
| Height (cm) | All | 27 | 173.09 (± 7.69) | 172.81 (± 7.70) | -0.28 |
| Body Mass (kg) | All | 27 | 85.06 (± 17.71) | 85.83 (± 18.29) | 0.77 |
| Body Mass Index (kg·m²) | All | 27 | 28.41 (± 5.61) | 28.73 (± 5.57) | 0.33 |
| Body Fat Percentage (%) | All | 26 | 24.76 (± 11.37) | 26.79 (± 10.57) | 2.03 |
| Fat Mass Index (kg/m²) | All | 26 | 7.34 (± 4.71) | 7.92 (± 4.53) | 0.58 |
| Fat-Free Mass Index (kg/m²) | All | 26 | 20.58 (± 2.24) | 20.27 (± 2.31) | -0.31 |
| Functional Reach Test (cm) | All | 26 | 37.34 (± 6.53) | 40.27 (± 5.96) | 2.93 |
| Sit-and-Reach Test (cm) | All | 26 | 27.53 (± 9.38) | 28.46 (± 8.36) | 0.93 |
| Cognitive Block Average (s) | All | 27 | 33.54 (± 3.04) | 31.61 (± 2.66) | -1.93 |
| Vertical Jump Height (cm) | All | 23 | 35.79 (± 7.96) | 35.24 (± 7.26) | -0.55 |
| Grip Strength (kg) | All | 27 | 14.26 (± 3.06) | 15.14 (± 3.02) | 0.88 |
| Isometric Midthigh Pull (kg) | All | 20 | 173.72 (± 66.40) | 176.72 (± 69.58) | 3 |
| Plank (s) | All | 25 | 155.32 (± 79.87) | 171.56 (± 82.74) | 16.24 |
| Estimated VO_2max_ (ml·kg^-1^·min^-1^) | All | 23 | 35.73 (± 7.70) | 34.78 (± 7.37) | -0.95 |
| ***Note:*** *Values are presented as mean (± SD). MD = Mean Difference (post – pre). TPP = Tailored Performance Plan. Positive values indicate increases from pre- to post-assessment, and negative values indicate decreases. Sample sizes (n) vary slightly by variable due to missing data. Units correspond to those shown in the Variable column.* | | | | | |

| **Supplementary Table 3: Participant Characteristics and Descriptive Statistics for Non-TPP Users** | | | | | |
| --- | --- | --- | --- | --- | --- |
| **Variable** | **Group** | **n** | **Mean (± SD)** | **Mean (± SD)** | **Mean_Diff** |
| Age | All | 205 | 36.7 (± 4.25) | 37.37 (± 4.35) | 0.67 |
| Height (cm) | All | 201 | 174.09 (± 9.91) | 174.07 (± 9.53) | -0.02 |
| Body Mass (kg) | All | 199 | 82.63 (± 14.09) | 83.75 (± 14.17) | 1.12 |
| Body Mass Index (kg·m²) | All | 199 | 27.29 (± 4.38) | 27.55 (± 4.66) | 0.27 |
| Body Fat Percentage (%) | All | 199 | 23.68 (± 8.24) | 26.21 (± 8.72) | 2.53 |
| Fat Mass Index (kg/m²) | All | 197 | 6.68 (± 3.2) | 7.38 (± 3.82) | 0.69 |
| Fat-Free Mass Index (kg/m²) | All | 197 | 20.59 (± 2.66) | 20.19 (± 2.54) | -0.4 |
| Functional Reach Test (cm) | All | 200 | 39.23 (± 5.51) | 42.72 (± 5.30) | 3.49 |
| Sit-and-Reach Test (cm) | All | 198 | 26.68 (± 9.78) | 27.89 (± 9.62) | 1.21 |
| Cognitive Block Average (s) | All | 200 | 32.47 (± 2.14) | 31.37 (± 2.53) | -1.1 |
| Vertical Jump Height (cm) | All | 190 | 38.72 (± 8.17) | 37.61 (± 8.33) | -1.11 |
| Grip Strength (kg) | All | 199 | 15.53 (± 3.18) | 15.59 (± 3.32) | 0.05 |
| Isometric Midthigh Pull (kg) | All | 159 | 204.32 (± 63.14) | 195.71 (± 63.68) | -8.61 |
| Plank (s) | All | 196 | 144.28 (± 57.58) | 150.6 (± 57.44) | 6.32 |
| Estimated VO_2max_ (ml·kg^-1^·min^-1^) | All | 189 | 36.07 (± 5.74) | 35.06 (± 5.81) | -1.01 |
| ***Note:*** *Values are presented as mean (± SD). MD = Mean Difference (post – pre). Non-TPP = No Tailored Performance Plan utilized. Positive values indicate increases from pre- to post-assessment, and negative values indicate decreases. Sample sizes (n) vary slightly by variable due to missing data. Units correspond to those shown in the Variable column.* | | | | | |

| **Supplementary Table 4: Comparison of Fitness Metrics by Sex in TPP Participants** | | | | | |
| --- | --- | --- | --- | --- | --- |
| **Variable** | **Group** | **n** | **Mean (± SD)** | **Mean (± SD)** | **Mean_Diff** |
| Age (yrs) | Male | 19 | 39.42 (± 7.12) | 39.95 (± 7.08) | 0.53 |
|  | Female | 8 | 36 (± 2.27) | 36.38 (± 2.20) | 0.38 |
| Height (cm) | Male | 19 | 176.56 (± 5.39) | 176.33 (± 5.41) | -0.23 |
|  | Female | 8 | 164.86 (± 5.94) | 164.46 (± 5.64) | -0.4 |
| Body Mass (kg) | Male | 19 | 85.23 (± 17.49) | 86.86 (± 18.65) | 1.63 |
|  | Female | 8 | 84.66 (± 19.42) | 83.4 (± 18.39) | -1.26 |
| Body Mass Index (kg·m²) | Male | 19 | 27.28 (± 5.01) | 27.87 (± 5.23) | 0.58 |
|  | Female | 8 | 31.08 (± 6.39) | 30.79 (± 6.17) | -0.29 |
| Body Fat Percentage (%) | Male | 18 | 18.72 (± 6.45) | 21 (± 5.18) | 2.28 |
|  | Female | 8 | 38.34 (± 7.47) | 39.83 (± 7.32) | 1.49 |
| Fat Mass Index (kg·m²) | Male | 18 | 5.14 (± 2.55) | 5.83 (± 2.31) | 0.69 |
|  | Female | 8 | 12.28 (± 4.79) | 12.61 (± 4.89) | 0.33 |
| Fat Free Mass Index (kg·m²) | Male | 18 | 21.37 (± 1.93) | 21.2 (± 1.96) | -0.17 |
|  | Female | 8 | 18.79 (± 1.90) | 18.16 (± 1.56) | -0.63 |
| Functional Reach Test (cm) | Male | 18 | 36.41 (± 6.90) | 40.78 (± 6.86) | 4.37 |
|  | Female | 8 | 39.44 (± 5.41) | 39.12 (± 3.25) | -0.31 |
| Sit-and-Reach Test (cm) | Male | 19 | 26.59 (± 9.42) | 27.37 (± 8.41) | 0.77 |
|  | Female | 7 | 30.07 (± 9.48) | 31.43 (± 8.06) | 1.36 |
| Cognitive Response Time (s) | Male | 19 | 32.91 (± 3.03) | 30.83 (± 2.54) | -2.08 |
|  | Female | 8 | 35.04 (± 2.68) | 33.47 (± 2.02) | -1.57 |
| Countermovement Jump (cm) | Male | 15 | 40.03 (± 5.85) | 39.29 (± 4.16) | -0.75 |
|  | Female | 8 | 27.84 (± 4.48) | 27.65 (± 5.48) | -0.19 |
| Grip Strength (kg/m²) | Male | 19 | 15.07 (± 3.13) | 16.24 (± 2.62) | 1.17 |
|  | Female | 8 | 12.35 (± 1.92) | 12.53 (± 2.27) | 0.18 |
| Isometric Midthigh Pull (kg) | Male | 12 | 204.52 (± 64.18) | 209.83 (± 61.89) | 5.31 |
|  | Female | 8 | 127.5 (± 37.80) | 127.04 (± 49.19) | -0.46 |
| Plank (s) | Male | 17 | 182.65 (± 78.23) | 183.59 (± 94.29) | 0.94 |
|  | Female | 8 | 97.25 (± 47.08) | 146 (± 45.31) | 48.75 |
| Estimated VO_2max_ (ml·kg^-1^·min^-1^) | Male | 15 | 39.49 (± 5.16) | 38.67 (± 4.86) | -0.82 |
|  | Female | 8 | 28.68 (± 6.79) | 27.48 (± 5.48) | -1.2 |
| ***Note:*** *Values are presented as mean (± SD). MD = Mean Difference (post – pre). TPP = Tailored Performance Plan. Positive values indicate increases from pre- to post-assessment, and negative values indicate decreases. Sample sizes (n) vary slightly by variable due to missing data. Units correspond to those shown in the Variable column.* | | | | | |

| **Supplementary Table 5: Comparison of Fitness Metrics by Sex in Non-TPP Participants** | | | | | | |
| --- | --- | --- | --- | --- | --- | --- |
| **Variable** | **Group** | **n** | **Mean (± SD)** | **Mean (± SD)** | **Mean_Diff** |  |
| Age (yrs) | Male | 146 | 36.47 (± 3.76) | 37.17 (± 3.90) | 0.71 |  |
|  | Female | 59 | 37.27 (± 5.25) | 37.86 (± 5.32) | 0.59 |  |
| Height (cm) | Male | 144 | 178.1 (± 7.47) | 178.07 (± 7.04) | -0.03 |  |
|  | Female | 57 | 163.95 (± 7.91) | 163.96 (± 7.25) | 0 |  |
| Body Mass (kg) | Male | 142 | 86.89 (± 12.01) | 88.25 (± 12.85) | 1.36 |  |
|  | Female | 57 | 72.02 (± 13.36) | 72.53 (± 13.30) | 0.51 |  |
| Body Mass Index (kg·m²) | Male | 142 | 27.44 (± 3.69) | 27.81 (± 3.34) | 0.37 |  |
|  | Female | 57 | 26.9 (± 5.76) | 26.91 (± 5.33) | 0.01 |  |
| Body Fat Percentage (%) | Male | 143 | 20.44 (± 5.97) | 22.87 (± 5.18) | 2.42 |  |
|  | Female | 56 | 31.94 (± 7.48) | 34.75 (± 7.46) | 2.81 |  |
| Fat Mass Index (kg·m²) | Male | 141 | 5.79 (± 2.25) | 6.5 (± 2.08) | 0.71 |  |
|  | Female | 56 | 8.92 (± 4.05) | 9.58 (± 4.24) | 0.66 |  |
| Fat Free Mass Index (kg·m²) | Male | 141 | 21.63 (± 2.06) | 21.33 (± 2.85) | -0.3 |  |
|  | Female | 56 | 17.95 (± 2.11) | 17.31 (± 2.56) | -0.64 |  |
| Functional Reach Test (cm) | Male | 142 | 39.72 (± 5.46) | 43.72 (± 5.14) | 4 |  |
|  | Female | 58 | 38.03 (± 5.50) | 40.26 (± 5.05) | 2.23 |  |
| Sit-and-Reach Test (cm) | Male | 140 | 24.53 (± 9.54) | 25.96 (± 9.28) | 1.43 |  |
|  | Female | 58 | 31.86 (± 8.34) | 32.54 (± 8.85) | 0.68 |  |
| Cognitive Response Time (s) | Male | 143 | 32.21 (± 1.98) | 30.97 (± 1.22) | -1.24 |  |
|  | Female | 57 | 33.11 (± 2.40) | 32.37 (± 2.95) | -0.74 |  |
| Countermovement Jump (cm) | Male | 137 | 41.82 (± 6.31) | 40.55 (± 6.92) | -1.27 |  |
|  | Female | 53 | 30.7 (± 6.91) | 30.01 (± 6.73) | -0.69 |  |
| Grip Strength (kg/m²) | Male | 144 | 16.7 (± 2.59) | 16.86 (± 2.60) | 0.16 |  |
|  | Female | 55 | 12.48 (± 2.47) | 12.25 (± 2.64) | -0.23 |  |
| Isometric Midthigh Pull (kg) | Male | 116 | 227.78 (± 54.42) | 219.15 (± 54.87) | -8.63 |  |
|  | Female | 43 | 141.02 (± 35.59) | 132.47 (± 35.34) | -8.56 |  |
| Plank (s) | Male | 143 | 151.18 (± 56.39) | 158.18 (± 56.41) | 7 |  |
|  | Female | 53 | 125.66 (± 57.17) | 130.13 (± 57.58) | 4.47 |  |
| Estimated VO_2max_ (ml·kg^-1^·min^-1^) | Male | 136 | 38.08 (± 4.81) | 36.95 (± 4.89) | -1.13 |  |
|  | Female | 53 | 30.89 (± 4.62) | 30.2 (± 4.12) | -0.7 |  |
| ***Note:*** *Values are presented as mean (± SD). MD = Mean Difference (post – pre). Non-TPP = No Tailored Performance Plan utilized. Positive values indicate increases from pre- to post-assessment, and negative values indicate decreases. Sample sizes (n) vary slightly by variable due to missing data. Units correspond to those shown in the Variable column.* | | | | | | |

| **Supplementary Table 6. Mixed-Effects Model Results for Pre–Post Fitness Changes Comparing Tailored Performance Plan (TPP) Users and Non-Users** | | | | | | | | | | | | |
| --- | --- | --- | --- | --- | --- | --- | --- | --- | --- | --- | --- | --- |
| Variable | Model 1 | | | | | | Model 2 | | | | | |
|  | Timepoint Estimate (S.E.) | *p*-value | TPP Estimate (S.E.) | *p*-value | Interaction Estimate (S.E.) | *p*-value | Timepoint Estimate (S.E.) | *p*-value | TPP Estimate (S.E.) | *p*-value | Interaction Estimate (S.E.) | *p*-value |
| BMI | 0.196 (0.147) | 0.184 | 0.676 (0.782) | 0.388 | 0.104 (0.418) | 0.804 | 0.214 (0.142) | 0.132 | 0.137 (0.158) | 0.387 | -0.021 (0.098) | 0.833 |
| Body Fat Percentage (%) | 2.405 (0.220) | 0.004 | 0.457 (1.118) | 0.683 | -0.455 (0.636) | 0.475 | 2.382 (0.211) | <0.001 | 0.422 (0.223) | 0.059 | -0.089 (0.148) | 0.548 |
| Fat Mass Index (kg/m²) | 0.646 (0.091) | 0.004 | 0.430 (0.503) | 0.393 | -0.094 (0.260) | 0.718 | 0.645 (0.087) | <0.001 | 0.165 (0.101) | 0.103 | -0.032 (0.061) | 0.595 |
| Fat-Free Mass Index (kg/m²) | -0.398 (0.087) | 0.004 | 0.063 (0.350) | 0.858 | 0.085 (0.252) | 0.735 | -0.388 (0.083) | <0.001 | -0.056 (0.070) | 0.429 | -0.003 (0.058) | 0.955 |
| Sit-and-Reach Test (cm) | 1.367 (0.254) | 0.004 | 0.999 (1.609) | 0.535 | -0.287 (0.725) | 0.693 | 1.252 (0.248) | <0.001 | -0.522 (0.400) | 0.192 | 0.307 (0.284) | 0.281 |
| Cognitive Block Average (s) | -1.242 (0.161) | 0.004 | 0.921 (0.429) | 0.032 | -0.812 (0.466) | 0.083 | -1.335 (0.156) | <0.001 | 0.122 (0.087) | 0.161 | -0.009 (0.108) | 0.934 |
| Vertical Jump Height (cm) | -0.807 (0.300) | 0.008 | -1.613 (1.072) | 0.133 | 0.442 (0.904) | 0.625 | -0.793 (0.289) | 0.007 | -0.482 (0.215) | 0.026 | 0.116 (0.199) | 0.559 |
| Grip Strength (kg/m^2^) | 0.117 (0.108) | 0.28 | -0.696 (0.450) | 0.123 | 0.737 (0.311) | 0.019 | 0.178 (0.105) | 0.091 | -0.147 (0.091) | 0.105 | 0.087 (0.073) | 0.239 |
| Lower Body Strength (kg_IMTP_/kg_WT_) | -0.135 (0.030) | 0.004 | -0.187 (0.103) | 0.07 | 0.136 (0.089) | 0.131 | -0.122 (0.029) | 0.004 | -0.062 (0.020) | 0.002 | 0.000 (0.020) | 0.983 |
| Plank (s) | 7.061 (2.929) | 0.017 | 13.483 (11.484) | 0.241 | 9.620 (8.639) | 0.267 | 7.985 (2.831) | 0.005 | -1.868 (2.287) | 0.415 | 0.370 (1.974) | 0.851 |
| Estimated VO_2max_ (ml·kg-1·min-1) | -0.851 (0.232) | <0.001 | 0.398 (0.889) | 0.655 | 0.078 (0.698) | 0.911 | -0.878 (0.224) | <0.001 | -0.347 (0.176) | 0.053 | 0.095 (0.152) | 0.533 |
| ***Note:*** *Values are presented as model estimates (standard error, S.E.) with corresponding p-values. Model 1 included timepoint (pre vs. post) as a fixed effect, a binary indicator for Tailored Performance Plan (TPP) participation (yes/no), and their interaction to test whether TPP users exhibited different pre–post changes than non-users. Model 2 replaced the binary indicator with a continuous measure of TPP session count (dose) and its interaction with timepoint to assess potential dose–response effects. All models controlled for sex and age, with participant ID included as a random intercept to account for within-subject variability. Positive estimates reflect increases from pre- to post-testing, while negative estimates reflect decreases.* | | | | | | | | | | | | |

***Supplementary Figure 1.*** *Standardized regression coefficients from multivariate multiple regression (MMR) models examining associations between survey-reported influences and post-program fitness outcomes. Each panel displays results for a distinct fitness measure. Predictors were ordinally scored (–1 = negative, 0 = neutral/not selected, 1 = positive) and standardized prior to analysis. Coefficients represent the change (in standard deviation units) in the outcome associated with a 1 standard deviation increase in the predictor score. Error bars reflect 95% confidence intervals. Asterisks (*) indicate coefficients whose confidence intervals do not include zero (p < 0.05). Coefficients are colored (red) to distinguish statistically significant from non-significant estimates.*
